# Surgery on the Trabecular Meshwork: Histopathological Evidence

**DOI:** 10.5005/jp-journals-10008-1184

**Published:** 2015-09-25

**Authors:** Shibal Bhartiya, Parul Ichhpujani, Tarek Shaarawy

**Affiliations:** Senior Consultant, Glaucoma Facility, Fortis Memorial Research Institute, Gurgaon Haryana, India; Associate Professor, Department of Ophthalmology, Glaucoma Facility, Government Medical College and Hospital, Chandigarh, India; Head, Glaucoma Sector, Geneva University Hospital, Rue Gabrielle-Perret-Gentil 4, 1205 Geneva, Switzerland

**Keywords:** Aqueous outflow and resistance, Histopathological basis of glaucoma surgery, Outflow facility, Trabecular meshwork.

## Abstract

Juxtacanalicular (JXT) trabecular meshwork and endothelial lining of Schlemm’s canal have been cited as the loci of aqueous outflow resistance, both in a normal as well as a glaucomatous eye. In this review, we attempt to understand the currently available surgical modalities in light of the available histopathological evidence, regarding localization of outflow resistance.

**How to cite this article:** Bhartiya S, Ichhpujani P, Shaarawy T. Surgery on the Trabecular Meshwork: Histopathological Evidence. J Curr Glaucoma Pract 2015;9(2):51-61.

## INTRODUCTION

In primary open angle glaucoma (POAG), the primary cause of increase to outflow resistance is located within the trabecular meshwork (TM), and logically may be eliminated by incising the same.^1-10^ In view of current knowledge, microsurgical dissection of the TM has again become the focus of attention among glaucoma surgeons.^7-8^ Even though there has been little addition in the existing knowledge about the classic procedures in terms of mechanism of action, there has been a trend toward more physiological approach, with regard to resistance to aqueous outflow in newer surgical approaches.^9^ This review is an attempt to analyze the currently available surgical modalities on the basis of available histopathological evidence regarding localization of outflow resistance.

### Functional Anatomy

The trabecular meshwork bridges the scleral sulcus, converting it into a circumferential channel, called the Schlemm’s canal (SC). The TM is a triangular, porous structure, in cross-section, that consists of connective tissue surrounded by endothelium. It is divided into three components: uveal meshwork, corneoscleral meshwork and juxtacanalicular (JXT) meshwork.^11,12^

The uveal meshwork forms the lateral border of the anterior chamber, extending from the iris root and ciliary body to the peripheral cornea. It consists of bands of connective tissue, with irregular openings that measure between 25 and 75 microns.

The corneoscleral meshwork extends from the sclera spur to the anterior wall of the scleral sulcus. It is the most extensive portion of the TM, and is composed of perforated sheets that become progressively smaller nearing SC.

The outermost part of the TM, composed of a layer of connective tissue lined on either side by endothelium, is called the JXT meshwork. The central connective tissue layer has variable thickness and is non-fenestrated, and the outer endothelial layer comprises the inner wall of SC.^11-17^

The outermost JXT or cribriform region has no colla-genous beams, rather several cell layers immersed in a loose web of extracellular matrix (ECM) fibrils, with small tortuous aqueous pathways that appear as empty space under electron microscopy. The ECM contains basement membrane material, proteoglycans, and glycosaminogly-cans, providing significant outflow resistance.^18^

The SC is comprised of endothelial cells surrounded by connective tissue, like a vein. This has the highest hydraulic conductivity of any endothelium in the body. It constitutes a leaky lining probably because of the numerous micron-sized transcellular pores in the endothelium and the associated giant vacuoles.^19,20-25^

Schlemm’s canal, under normal conditions, is not a site of considerable outflow resistance. However, with an increase in intraocular pressure (IOP), the TM expands into the lumen of the canal, causing its concomitant narrowing, with a significant increase in outflow resistance.^25^ Collagenous septae between the inner and outer walls, especially near the collector channels, are probably a safeguard against collapse of the canal and occlusion of collector channels with an increase in IOP.^25,26^

The SC possesses internal collector channels and is connected to episcleral and conjunctival veins through the external collector channels, the intrascleral venous plexus, the deep scleral plexus and the aqueous veins.^27,28^

### Aqueous Outflow and Resistance

Almost 70% aqueous humor outflow is accounted for by the ‘conventional’ outflow pathway via the anterior chamber to the trabecular meshwork, SC, and collector channels; entering the systemic venous circulation in the episcleral veins. The remaining 30% may leave the ‘eye through the uveoscleral, or unconventional’ outflow pathways; which is a passive fluid movement down a pressure gradient.^19,29-31^

In uveoscleral outflow, aqueous humor enters the ciliary muscle and exits through the supraciliary space, across the anterior or posterior sclera through the emissarial canals around the vortex veins, or into the choroidal vessels.^32,33^

Uveoscleral outflow may be considered analogous to lymphatic drainage of tissue fluid, as the fluid may mix with tissue fluid from the ciliary muscle, ciliary processes and choroid; and is drawn osmotically into the veins.^34^ In non-human primates, 40 to 50% of aqueous humor exits the eye by the uveoscleral route. In human eyes, most data have been collected by indirect calculations, using the expanded Goldmann equation [F = (Pi - Pe) × C + U; where, ‘F’ is the rate of aqueous humor formation, ‘Pi’ is the intraocular pressure, ‘Pe’ is the episcleral venous pressure, ‘C’ is the tonographic facility of outflow, and ‘U’ is the pressure insensitive parameter to symbolize uveoscleral outflow], suggesting a similar fraction, at least in eyes, from younger individuals.^35,36^

Ciliary muscle contraction greatly affects uveoscleral outflow, and prostaglandin F_2α_ greatly increases uveoscleral outflow by decreasing the flow resistance of the interstitial spaces in the ciliary muscle.^37,38^

In eyes treated with atropine, uveoscleral flow accounts for 4 to 27% of the total outflow, but with pilocarpine it was only 0 to 3%.^39^ An age-dependent reduction in uveoscleral flow in human eyes may explain the initial difference seen between non-human primate and human eyes.^11,40^

### Localization of Resistance

In humans, 75% of the resistance to the aqueous humor outflow is localized to the TM, and 25% beyond SC, in the outer wall of SC or tissue surrounding it.^2,9,11,41-48^ The major site of resistance within the TM structure has not yet been well characterized, but most investigators agree that almost two thirds (up to 75%) is localized in the JXT portion.^2,9,42,43,46,49^ There have been reports that the incision of the uveal and proximal corneoscleral layers of the TM did not affect outflow resistance, while a deeper incision through the entire meshwork and into SC, eliminated 75% of the normal outflow resistance.^2,9^

The major mechanisms through which resistance across the conventional outflow pathway may be regulated are:

- Transcellular route through vacuoles in the inner wall endothelial cells of SC.^50-54^
- Paracellular route passing between SC inner wall endothelial cells.^54-57^
- Turnover of extracellular matrix in the JXT region.^54,58-64^

It is important to note that aqueous can only reach either transcellular or paracellular pathways after traversing the JXT region of TM.^54^

Outflow resistance within the ciliary body (CB) is postulated to be regulated through ECM turnover in CB stroma and sclera, as well as by ciliary body smooth muscle cell tone, although the latter is controversial.^24,54,65-71^ Thus, both restructuring and turnover of ECM regulate outflow resistance in both conventional and uveoscleral pathways, through unknown molecular mechanisms. The ciliary muscle moves in an anterior and inward direction, resulting in spreading of the TM and dilation of SC during its contraction, thus decreasing outflow resistance. The opposite occurs during relaxation with a consequent increase in outflow resistance.^11,72^ Voluntary accommodation, electrical stimulation of the trigeminal nerve and local or systemic administration of cholinergic agents have been shown to decrease outflow resistance in animal models.^73-77^

### Aqueous Outflow Resistance and Glaucoma

The outflow system responds biomechanically to the IOP, with IOP increase resulting in tissue and cellular deformation at the level of the SC endothelium. These pressure and shear stress-mediated signals initiate a highly complex interactive network of cellular, molecular and genetic changes, inducing both rapid responses and slow adaptive changes that regulate pressure and flow resulting in long-term homeostasis.^11,78-80^

A failure of this homeostatic mechanism results in chronic IOP increase, and the consequent characteristic optic neuropathy called ‘glaucoma’. The increased IOP found in glaucoma is caused by an increase in aqueous outflow resistance within the drainage pathways and not by excess secretion of aqueous humor.^9,20,81^

Alterations in the trabeculum are presumed to lead to increased aqueous humor outflow resistance and elevated IOP in patients with POAG. Loss of trabecular cells is presumed to cause the structural alterations in the trabeculum. These changes are trabecular thickening, fusion of trabecular beams and accumulation of extracellular material in the endothelial meshwork, as well as a decrease in trabecular meshwork cellularity.^82-89^

There are several hypotheses pertaining to decreased trabecular cellularity in glaucoma.^88-99^ Grierson’s hypothesis of migration of trabecular cells from the trabeculae to the endothelial meshwork in early POAG can explain the ‘activated’ and enlarged trabecular cells seen in the endo-thelium meshwork.^90^

Supranormal phagocytic activity of trabecular cells may be responsible for cell death,^94^ as well the detachment of cells from the meshwork and migration toward the SC.^94-96^ Mechanical stress resulting from elevated IOP or through trabecular hypoperfusion may also be contributory factors for this loss of trabecular cellularity.^90,92^

The ECM thickness beneath the inner wall of SC and in the JXT meshwork has been found to be higher in glaucomatous eyes than age-matched healthy con-trols.^11,97,98^ Modifications of the trabecular matrix with plaque like trabecular thickening and fusion, accumulation of ECM in the endothelial meshwork, atypical collagen and abundance of long spacing have also been reported.^89,97,100-103^ An impairment of the endothelial wall of SC in areas of modified cribriform extracellular matrix has also been seen.^92,100^

Biochemical analyses of TM specimens and aqueous humor proteins have reported a variety of differences between normal and glaucomatous eyes at the molecular level. Levels of TGF-beta2, VEGF, endothelin, PAI, and soluble CD44 are known to be elevated in the aqueous humor of POAG as compared to normal aqueous humor. The ECM is reported to have less hyaluronic acid and more chondroitin sulfate.

These molecules may be the reason for the changes seen in the TM, but it is essential to keep in mind that these may be attributable to the chronic use of glaucoma medications, the effect of which on the molecular composition of TM is yet to be ascertained.^104-112^

The collapse of SC may be considered contributory to the increase in outflow resistance. However, outflow resistance at high IOPs has not been found to be as high as those found in glaucoma. In normal eyes, facility of outflow decreases from 0.40 μL/min/mm Hg at 10 mm Hg to 0.28 μL/min/mm Hg at 50 mm Hg. In comparison to this, the facility of eyes with POAG is usually less than 0.13.^19,26,113^

### Bypassing the Resistance

Most current surgical modalities attempt to lower the IOP by increasing aqueous outflow, but not for cycloablative procedures that endeavor to decrease aqueous humor production.

Surgeries, such as trabeculectomy, glaucoma drainage implants and shunts (which penetrate the TM and cannulate SC or create a path through the scleral wall), all attempt to bypass outflow resistance by shunting aqueous humor away from the TM. Canaloplasty and newer shunts which create a mini-cyclodialysis cleft increase the uveoscleral outflow by opening existing aqueous drainage pathways or creating new pathways.

Deep sclerectomy unroofs the SC, removing its inner wall as well the JXT meshwork, removing the site of maximal outflow resistance.

Surgeries that Increase Aqueous Outflow

1. Surgeries that bypass conventional aqueous outflow pathway:
  - Trabeculectomy
  - Non-penetrating deep sclerectomy
  - Glaucoma drainage devices
  - Viscocanalostomy, Canaloplasty
  - Ab interno trabeculotomy or Trabectome surgery
  - Trabeculotomy (limited to congenital glaucoma)
  - Goniotomy (limited to congenital glaucoma)
2. Surgeries that increase the uveoscleral outflow:
  - Suprachoridal Gold SOLX shunt
  - CyPass
  - Express shunt
  - I stent

Surgeries that Decrease Aqueous Production

- Contact (transcleral) cycloablation
  – Cyclocryotherapy
  – Diode
  – Nd:YAG
- Noncontact cycloablation
  – Nd:YAG
  – Diode
- Transpupillary argon green cyclophotocoagulation
- Endolaser ablation
  – Diode
  – Argon

This review focuses on the surgeries on the trabecular meshwork, all of which work on the principle of increasing aqueous outflow.

### Surgeries that Increase Aqueous Outflow

The era of modern surgery for glaucoma was heralded by the introduction of the concept of trabeculotomy by Smith, which was developed further by Harms and Dannheim.^114,115^ Krasnov introduced sinusotomy when trabecular function appeared adequate.^116^ Sinusotomy and trabeculotomy were combined to produce a filtering bleb with an intact anterior chamber.^117^ In 1968, Cairns introduced the current gold standard of glaucoma surgery, the ‘trabeculectomy’.^118^

Mechanism of Action

*Trabeculectomy:* Trabeculectomy essentially functions as a guarded full thickness sclerectomy, although Cairns originally postulated that removal of TM would allow free flow of fluid into the open lumen of the SC, bypassing trabecular resistance.^118^

A fistula between the anterior chamber and the subcon-junctival space directs the aqueous to the subconjunctival space, and is thereafter directly absorbed into the sclera and episcleral vasculature to enter the orbital circulation, bypassing both the conventional and uveoscleral pathways.^119^ A new pathway of outflow is thus created through the sclera and into a tissue not normally exposed to eye fluid pressure, fluid shear or tissue swelling.

Despite modulation of the subconjunctival space into a porous matrix, the procedure is not a physiological bypass, and remains dependent on the size of the ostium, tension in the sclera flap, as well as wound healing and its modulation.

A decrease in the hydraulic conductivity of the bleb capsule leads to a rise in fluid pressure within the bleb, changing its mechanical and biochemical environment leading to progressive scarring and consequent bleb failure.^119^

McEwen postulated that a single patent hole of 12 urn is by itself sufficiently large to provide a normal facility of outflow.^120^ A small sclerostomy (0.5 mm) has been found to be adequate, minimize astigmatism and the chance of limbal aqueous flow, and may maximize the chance of controlling outflow.^121^

Experiments have shown that the simple aqueous outflow of protruding human eyes increases 26 folds, from 0.24 ± 0.08 ml/min/mm Hg to 6.33 ± 6.67 ml/min/ mm Hg after external trabeculectomy. This may be explained by the fact that during external trabeculectomy, the diaphragm through the aqueous outflow consists only of the uveal meshwork and the largest inner part of the corneoscleral TM.^119^

The mean outflow facility after non-penetrating glaucoma surgeries (NPGS) (1.584 ± 0.217 μl/min/mm Hg) is relatively lower than reported mean outflow facility after trabeculectomy (2.96 ± 0.60 μl/min/mm Hg), which possibly accounts for the gradual decrease in IOP after NPGS, opposed to the sudden drop in pressure after penetration as in the case of trabeculectomy.^122,123^

*Schie’s procedure or full thickness filtration surgeries:* Full thickness procedures like thermal sclerostomy, anterior or posterior lip sclerectomy and Elliots’ trephination lack a guard over the sclerostomy except the tenons-conjunctival complex, with a limbus-based conjunctival flap. The tamponading effect of the partial thickness sclera flap is lacking and the aqueous egress is unimpeded.

They are therefore prone to problems relating to hypotony and over filtration, cataract formation due to shallow ACs as well as late infections, and hence are no longer performed.

*Shunts/Tubes:* All commercially available conventional shunts consist of a tube designed to shunt aqueous from the anterior chamber to a distal plate in the posterior subconjunctival space, from where aqueous is directly absorbed into the sclera and episcleral vasculature to enter the orbital circulation, bypassing both the conventional and uveoscleral pathways. The primary tube-plate junction includes a rim, through which the tube empties onto the explant plate surface to avoid closure of the tube orifice following eventual encapsulation of the device by fibrosis. The shunting to the metabolically less active posterior subconjunctival filtration has implications in terms of potential advantages, such as less subconjunctival fibrosis, larger subconjunctival reservoir and less bleb dysthesias and failure rates.

Examples of aqueous shunts include the―Ahmed (New World Medical Inc, Rancho Cucamonga, CA), Baerveldt (Advanced Medical Optics Inc, Santa Ana, CA), Krupin (Eagle Vision) and Molteno (Molteno Ophthalmic Limited, Dunedin, New Zealand) shunts. These devices differ depending on explant surface areas, shape, plate thickness, the presence or absence of a valve and details of surgical installation.

Incorporation of a valve or flow restrictor in aqueous shunts, in theory, should reduce the risk of immediate postoperative hypotony common to nonvalved devices when placed without temporary tube ligation.^124-126^

*Non-penetrating glaucoma surgeries:* Non-penetrating surgeries are based on the premise that aqueous egress occurs at the level of SC and its efferents, and that the selective removal of the external part the trabecular meshwork that is mainly involved in aqueous outflow resistance (inner wall of SC and the adjacent trabecular meshwork), while leaving intact the innermost trabecular meshwork layers.^11,127-130^ Thus, the outflow facility is increased, while retaining a degree of residual outflow resistance by leaving a membrane between the anterior chamber and the scleral dissection. This membrane is formed by the anterior and posterior trabecular mesh-work, the internal endothelium of Schlemm’s canal and Descemet’s membrane in deep sclerectomy or viscocanalostomy.^119^

The removal of the JXT trabecular meshwork and the inner wall of SC to decrease aqueous outflow resistance was proposed by Zimmerman et al.^128^ This procedure was called ‘ab-externo trabeculectomy’, and performed under a scleral flap. Drainage was found to occur at the level of the posterior trabeculum, with an increase in the outflow facility following removal of the inner wall of SC and the JXT meshwork (from 0.2 ± 0.6 to 2.03 ± 1.43 μl/ min per mm Hg).^127-131^

Deep sclerectomy consisted of removal of corneal stroma behind the anterior trabeculum and the Descemet’s membrane under a scleral flap, so that aqueous humor drainage occurred at the level of the anterior trabeculum and Descemet’s membrane.^132^ This was reported to result in a greatly increased outflow facility (from 0.19 ± 0.03 to 24.5 ± 12.6 μl/min per mm Hg).^133^

Modern day non-penetrating deep sclerectomy combines both of these procedures, and its complete nomenclature therefore is ‘deep sclerectomy with external trabeculectomy’.^127,132-141^

Viscocanalostomy is known to work on the same principle, but involves the additional injection of visco-elastic substance in the SC ostia.^143^ The procedure requires careful scraping of the bed of SC with a forceps, or trabecular aspiration. This leads to the removal of a homogenous external trabecular membrane in one coherent plane that allows aqueous humor to egress through the remaining inner trabecular layers.^127-144^

### Pathways of Aqueous Drainage in NPGS

Demoting Schlemm’s Canal

In external trabeculectomy, the removal of the inner wall of SC and the adjacent trabecular layers are designed to remove the part of the TM that is involved in the main aqueous outflow resistance. Removal of the outer wall causes damage to the inner wall of the canal as the septae, which bridge the inner and outer walls, are pulled away during the unroofing procedure. Such damage to the inner wall and adjacent JXT region effectively removes these regions and allows aqueous humor access to the canal.^19,20,129,145^ Other contributing factors include thinning of the trabecular meshwork and vaulting of residual trabecular meshwork vaults towards the intra-scleral cavity, leading to a widening of the cribriform interspace (similar to laser trabeculoplasty).^146^

*Ex-vivo* confocal microscopy has revealed that this external trabecular membrane involves not only the inner wall of SC and the JXT meshwork, but also a part of the corneoscleral layers.^138^ The drainage thus occurs at the level of the posterior trabeculum through the innermost layers of the TM, which are left intact and aqueous humor reaches the scleral lake^19,20,128^ and then the subconjunctival spaces as demonstrated by the presence of filtering bleb (observed in most of the cases of deep sclerectomy with external trabeculectomy; usually more diffuse or smaller than after trabeculectomy.^127^

Creation of Descemet’s Window

Excising a deep layer of sclera and exposing Descemet’s membrane may create a route for aqueous drainage that bypasses the meshwork, through the intact trabe-culodescemetic membrane namely at the level of the anterior trabeculum, from where it reaches the scleral lake.^19,20,128,132^ Descemet’s membrane, however, is not permeable enough to relieve the elevated pressure of glaucoma.^23,24^ A window of exposed Descemet’s membrane approximately 21 x 21 mm would be required to lower IOP to the low teens. Following its partial removal during the procedure, its permeability increases. Also, the permeability of the trabeculo Descemet’s membrane probably also increases due to properties of the meshwork remaining after the unroofing of SC.

An index of relative impermeability of Descemet’s membrane is supported by 41% goniopuncture rate after deep sclerectomy.^5^

Injection of Viscoelastic Material

In viscocanalostomy, injection of viscoelastic material into the ends of SC is designed to enhance aqueous egress through the cut ends of SC and through previously non-functional areas of SC, and then aqueous collector channels, thereby lowering IOP.^1^ The viscoelastic is resorbed in 4 to 5 days, which is not long enough to prevent healing of the cut ends of the canal. A more likely explanation is that expansion of the canal ruptures both the inner and outer endothelial walls of the canal, extending into the juxtacanalicular connective tissue and some of the meshwork.^22^ Therefore, both viscocanalostomy and deep sclerectomy function as ‘gentle’ trabeculotomies, allowing aqueous to bypass the site of abnormal outflow resistance, the JXT; through inadvertent ruptures in the JXT and the inner wall of the canal and also through the unroofed outer wall. If the ruptured regions of the JXT and canal heal with time, surgery may fail in eyes that did not develop filtration blebs.

### Alternate Pathways

Injection of viscoelastic in SC also results in focal disruptions of the inner wall endothelium of SC and disorganization of the JXT zone, resulting in direct communication of the JXT extracellular spaces with the lumen of SC. This may, therefore, initially enhance conventional aqueous outflow, accounting for approximately 30% increase in outflow facility in non-human primates.^147^ Disruption of the posterior wall of the SC may also provide direct communication between its lumen and the tissues of the CB, thereby enhancing uveoscleral outflow.^148^

The viscoelastic material also has anti-inflammatory properties and may inhibit cellular migration, phagocytosis and cytokine production; and thus may interfere with wound healing.^81^

After aqueous humor passage through the trabeculo-Descemet’s membrane, four hypothetical mechanisms of aqueous resorption may occur―a subconjunctival filtering bleb, an intrascleral filtering bleb, a suprachoroidal filtration and an episcleral vein outflow via Schlemm’s canal.^149,150^

### Canaloplasty

This is a new surgical procedure in which a tensioning suture is placed within the SC when possible, to apply inward directed tension on the TM, in addition to visco-dilation. Potentially, the suture tension may increase TM permeability, similar to the action of pilocarpine, as well as help maintain a patent canal lumen, similar to the intraocular tensioning suture.^12-14^

### Anterior Chamber Drainage Angle Surgery: Trabeculotomy, Trabeculectomy *ab interno*

Reported success of trabeculotomy and goniotomy has ranged between 68 and 100%, in infants and young children with congenital glaucoma. Poor long-term success in adults has been presumably due to the formation of anterior synechiae in the postoperative phase. Ab interno trabeculectomy using the Trabectome™ (NeoMedix, Tustin, CA) aims to selectively remove the TM and inner wall of SC, while leaving the rest of the outflow system (outer wall of SC, collector channels, and aqueous veins) relatively intact, and attempts to avoid anterior synechiae formation or other forms of wound healing with resultant closure of the cleft.^151-153^

A strip of TM and inner wall of SC spanning 80 to 100° is ablated and removed under direct gonioscopic visualization. Intraoperative reflux of blood through the resulting cleft is desirable in this procedure and confirms appropriate ab interno ‘unroofing’ of Schlemm’s canal. The simultaneous aspiration of tissue debris, theoretically, reduces the inflammatory stimuli and opportunity for scarring among shards of incised or ruptured tissues remaining after traditional goniotomy or trabeculotomy.

Importantly, it does not preclude standard surgery subsequently, as the conjunctiva is not traumatized; however, the IOP lowering effect has been less as compared to conventional filtration surgeries.

### Transtrabecular Shunt: iStent Trabecular Micro-Bypass Device

The iStent trabecular microbypass (Glaukos, Laguna Hills, Calif.) provides a channel for direct transtrabe-cular aqueous outflow from anterior chamber to collector channels. It is self-retaining and constructed of implant grade titanium Ti6AL4V and heparin coated implant, angled on one side of its arch shaped body, with an inlet of 80 mm internal diameter on the other side.

The iStent is placed via an ab externo approach under gonioscopic control into the SC.^154,155^ This microbypass stent sits in the canal itself, with a circumferential extension of 1 mm into the 36 mm long canal, with a snorkel in the anterior chamber through which the aqueous enters the SC.

Cultured autopsy eye perfusion experiments have shown that adding successive bypass shunts produces a step-wise increase in outflow. This *in vitro* experiment demonstrated a potential for achieving very low IOP with such devices, but is yet to be replicated *in vivo.* Reportedly, the first shunt has the most effect, dropping IOP from 21.4 ± 3.8 to 12.4 ± 4.2 mm Hg. Successive addition of up to four stents placed into SC produced step-wise reduction in system pressure (from 13.6 ± 4.1 to 10.0 ± 4.3 mm Hg); while complete removal of the meshwork lowered IOP to 6.3 ± 3.2 mm Hg.^156^

### EyePass Bi-Directional Glaucoma Implant

The EyePass Glaucoma Implant (GMP Companies, Inc, Ft Lauderdale, FL) is a bidirectional shunt that diverts aqueous from the anterior chamber directly into Schlemm’s canal. It consists of a dual 6.0 mm long silicone tube, bonded at one end for less than 1.0 mm, creating a Y-shape. The inner diameter of the silicone tube is 125 um and the outer diameter is 250 um, making the tube narrow enough to fit the lumen of the SC. The bidirectional shunt is easily identified after implantation as blood refluxes from Schlemm’s canal into the lumen of the unit. Not much has been reported about this device yet as it is undergoing clinical trials.

### Suprachoroidal Shunt

The solx gold shunt is an *ab externo* suprachoroidal trans-limbal shunt and works as a ‘controlled’ cyclodialysis, draining aqueous into the suprachoroidal space. The anterior end of this 24-karat gold device with dimensions of 5.2 mm length, 3.2 mm width and 44 to 68 mm thickness (XGS-5 and XGS-10, respectively) is placed into the anterior chamber over the sclera spur via a scleral incision and the posterior end positioned in the suprachoroidal space. It includes several channels through its body, besides those initially functioning that potentially can be successively opened after installation via a laser applied to windows in its anterior chamber component for *in vivo* postoperative adjustments in outflow, to compensate for possible decrease in outflow with time. The flow resistance is stated to be 0.65 to 1.3 mm Hg/ml/min. Aqueous flows both through the channels in the body of the shunt as well as around its body.

Mastropasqua et al used *in vivo* confocal microscopy to show that successful gold microshunt implantation significantly increased conjunctival microcysts density and surface at the site of the device insertion.^157^ These findings suggest that the enhancement of the aqueous filtration across the sclera may be one of the possible outflow pathways exploited by the shunt.

### EX-PRESS Glaucoma Filtration Device

EX-PRESS Glaucoma Filtration Device® (Alcon, Fort Worth, Texas) is an implantable stainless steel 2 to 3 mm long and 0.4 mm diameter tube, which connects the anterior chamber to the intrascleral space. The 27 gauge shaft is designed to approximate the thickness of the human sclera, and has a spur on its underside to prevent extrusion out of the anterior chamber (AC), and a plate on the scleral side to prevent implant migration into the AC. The tip has multiple orifices for aqueous egress, with a 50 um lumen which offers some resistance to aqueous flow, and a notch in the back that helps direct aqueous flow posteriorly. Its distal tip penetrates into the AC, while its proximal end is located under the scleral flap. The EX-PRESS glaucoma filtration device controls IOP by allowing a limited outflow of aqueous humor into the intrascleral space and thereafter into the subconjunctival space. The device is implanted underneath a partial thickness scleral flap through a needle sclerostomy, providing a more elegant and standardized sclerostomy than that of trabeculectomy. The EX-PRESS device-assisted guarded filtration procedure carries no risk of iris prolapse or bleeding from the ciliary body, unlike traditional trabe-culectomy.

## CYPASS MICRO-STENT

The CyPass is a micro-implantable polyimide device, 6 mm in length and a small lumen of 300 mm, which is placed in the supraciliary and suprachoroidal space to increase uveoscleral outflow by creating a small cyclo-dialysis. It allows for an ab interno surgical approach, and is implanted in the suprachoroidal space through a clear corneal incision, leaving the trabecular meshwork intact. The distal end of the device penetrates into the suprachoroidal space, while the proximal collar remains in the anterior chamber, kept in place by three rings on its collar.

Initial anecdotal reports are hopeful of its efficacy; and combination CyPass and cataract surgery trial (COMPASS) and CyPass clinical evaluation trial (CYCLE) are non-randomized multi-center trials currently underway to assess the CyPass.

Another trial, ‘DUETTE’, is a European trial evaluating two different versions of the CyPass in a prospective and randomized study.

## CONCLUSION

The quest for a more predictable and physiologic glaucoma procedure with rapid recovery and a greater margin of safety is ongoing. Even though encouraging results have been reported with non-penetrating glaucoma surgical procedures, it will take a while for glaucoma surgeons across the globe to adopt NPGS.
